# Prognostic significance of crazy paving ground grass opacities in non-HIV *Pneumocystis jirovecii* pneumonia: an observational cohort study

**DOI:** 10.1186/s12890-019-0813-y

**Published:** 2019-02-21

**Authors:** Shogo Kumagai, Machiko Arita, Takashi Koyama, Takao Kumazawa, Daiki Inoue, Atsushi Nakagawa, Yusuke Kaji, Kenjiro Furuta, Motonari Fukui, Keisuke Tomii, Yoshio Taguchi, Hiromi Tomioka, Tadashi Ishida

**Affiliations:** 1 Department of Respiratory Medicine, Kurashiki Central Hospital, 1-1-1 Miwa, Kurashiki, Okayama, 710-0052 Japan; 2 Department of Diagnostic Radiology, Kurashiki Central Hospital, Kurashiki, Okayama, Japan; 3 Respiratory Disease Center, Medical Research Institute, Kitano Hospital, Osaka, Japan; 4 Department of Respiratory Medicine, Kobe City Medical Center General Hospital, Kobe, Hyogo Japan; 5 Department of Respiratory Medicine, Tenri Hospital, Tenri, Nara Japan; 6 Department of Respiratory Medicine, Kobe City Medical Center West Hospital, Kobe, Hyogo Japan

**Keywords:** *Pneumocystis jirovecii* pneumonia, Ground grass opacities, Computed tomography

## Abstract

**Background:**

In patients with non-HIV *Pneumocystis jirovecii* pneumonia (PjP), computed tomography imaging reveals ground grass opacities (GGO). Previous reports show that some patients with non-HIV PjP exhibit GGO with crazy paving. However, there have been no studies on the association between crazy paving GGO and non-HIV PjP clinical outcomes. Here, at the diagnosis of non-HIV PjP, we reviewed high-resolution computed tomography (HRCT) findings that included GGO types and evaluated the prognostic impact of crazy paving GGO on the clinical outcomes of non-HIV PjP immunocompromised patients.

**Methods:**

We retrospectively reviewed the clinical information including the HRCT findings of patients diagnosed with non-HIV PjP from five institutions between 2006 and 2015. The GGO types included those with or without crazy paving. The associations between clinical factors such as HRCT findings and in-hospital mortality were assessed using the Cox regression model.

**Results:**

Sixty-one patients were included in our study. Nineteen patients died at a hospital. All patients exhibited GGO on HRCT imaging at diagnosis of non-HIV PjP. The HRCT findings included crazy paving GGO (29 patients, 47.5%), consolidations (23 patients, 37.7%), bronchiectasis (14 patients, 23.0%), and centrilobular small nodules (30 patients, 49.2%). Cysts were not observed in any patient. Multivariate analysis revealed that crazy paving GGO and low serum albumin levels were independent risk factors for mortality.

**Conclusions:**

At the diagnosis of non-HIV PjP, patients with crazy paving GGO on HRCT imaging and low serum albumin levels may have a poor prognosis.

**Electronic supplementary material:**

The online version of this article (10.1186/s12890-019-0813-y) contains supplementary material, which is available to authorized users.

## Background

*Pneumocystis jirovecii* causes common and life-threatening pneumonia in immunocompromised patients with or without human immunodeficiency virus (HIV) infection [1]. Non-HIV immunocompromised patients includes those with malignancies, those who experienced solid organ or bone marrow transplantations, and those with autoimmune diseases, connective tissue diseases, or inflammatory disorders treated with corticosteroids or immunosuppressants [1, 2]. Patients with *Pneumocystis jirovecii* pneumonia (PjP) present such symptoms as progressive dyspnoea, non-productive dry cough, and a light fever [1]. The clinical pictures of non-HIV PjP differ from those of HIV PjP [2–5], and the prognosis of non-HIV PjP is worse than that of HIV PjP. To prevent rapid progression towards severe respiratory failure and death, early diagnosis of PjP and the initiation of proper treatment is necessary [6]. The diagnosis of non-HIV PjP depends on the clinical history of the immunocompromised host, respiratory symptoms, radiographic findings, and microscopic examinations of or DNA detection in sputum or bronchoalveolar lavage fluid (BALF) [7]. High-resolution computed tomography (HRCT) is thought to be the most reliable imaging technique in detecting and differentiating pneumonias in immunocompromised patients [8, 9], and HRCT findings largely affect the utility of other diagnostic methods, including bronchoalveolar lavage (BAL). Several studies have evaluated HRCT findings during the diagnosis of non-HIV PjP and show that non-HIV PjP patients mainly exhibit ground grass opacities (GGO) on HRCT imaging [10–14]; some have GGO with superimposed interlobular septal thickening and intralobular lines (referred to as “the crazy paving appearance”) [7, 12]. However, few studies have investigated the clinical significance or impact on outcomes of HRCT findings that include crazy paving GGO.

Here, we describe the HRCT findings in non-HIV PjP patients and evaluate differences in the clinical characteristics and prognosis of patients with or without crazy paving GGO.

## Methods

### Patients

From June 2006 to December 2015, we included patients admitted to five participating institutions (Kurashiki Central Hospital, Kitano Hospital, Kobe City Medical Center General Hospital, Tenri Hospital, Kobe City Medical Center West Hospital) who were diagnosed with PjP. The Ethics Committees of the five participating institutions approved the present study. The patients included in our study met the following five inclusion criteria: (1) presence of an underlying immunodeficiency known to be associated with PjP; (2) clinical symptoms suggestive of a lower respiratory tract infection, i.e., cough or dyspnoea; (3) new pulmonary infiltrates on HRCT; (4) the presence of *P. jirovecii* DNA based on polymerase chain reaction (PCR) detection in a BALF or sputum sample with or without a positive direct staining result; and (5) significantly elevated plasma (1–3)-β-D-glucan (β-D-glucan). We excluded patients who were not administered a chest HRCT at diagnosis, were infected with HIV, had other concurrent infections, or had pulmonary comorbidities including interstitial pneumonia or lung cancer.

### Data collection

Patient demographic, clinical, radiographic, and laboratory data were retrospectively reviewed and collected. These data included the patient age; gender; smoking history; underlying diseases such as autoimmune or collagen vascular diseases, malignancies, and renal diseases; renal transplantation; corticosteroid or immunosuppressant use; trimethoprim/sulfamethoxazole (TMP/SMX) prophylaxis; initial symptoms; initial laboratory findings, including the PaO_2_/FiO_2_ (P/F) ratio and levels of lactate dehydrogenase (LDH), Krebs von den Lungen-6 (KL-6), and β-D-glucan; diagnostic methods (BALF or sputum); BALF findings; treatments; adjuvant corticosteroid treatment; mechanical ventilation; and in-hospital mortality.

### BAL procedures

After topical anaesthesia of the oropharynx with lidocaine, a fibre optic bronchoscope was passed into the airways and wedged in the bronchial tree where the abnormality on the HRCT was most apparent. BAL samples were obtained by instilling three 50-mL aliquots of warm saline solution (0.9%), followed by gentle suction after the infusion of each aliquot. A PCR assay and Giemsa and methenamine silver staining were performed to detect *P. jirovecii* in BALF samples. Laboratory processing of BALF included staining and culture for bacteria (i.e., mycobacteria) and fungi. For cytomegalovirus (CMV) detection, the samples of BALF were inoculated into shell vial cultures of human foetal lung fibroblasts and their growth was examined 1 and 7 days later using direct immunofluorescence staining.

### Thoracic HRCT

Computed tomography (CT) scans were obtained during the diagnosis of non-HIV PjP at each institution. All scans were reconstructed with a 1 or 2-mm slice thickness (2 mm at the Tenri Hospital, and 1 mm at the other four institutions) and viewed at the standard mediastinal windows (level, 35 Hounsfield units [HU]; width, 450 HU) and lung windows (level, − 700 HU; width, 1500 HU). All CT scans were sent to the central data centre for evaluation by two radiologists who were blinded to the clinical information, such as the underlying diseases. Evaluation decisions were based on consensus. The criteria to determine the density, pattern, and distribution of the infiltrates were as follows: (1) GGO were an area of hazy increased attenuation without obscuration of underlying vascular markings; (2) crazy paving GGO were GGO with superimposed interlobular septal thickening and intralobular lines; (3) non-crazy paving GGO were GGO without superimposed interlobular septal thickening and intralobular lines; (4) a mosaic distribution showed more or less sharply demarcated regions of different densities within the infiltrated lung parenchyma or regions of nearly uninvolved secondary lobules in between the infiltrates; (5) a peripheral sparing distribution predominantly showed a GGO area that did not reach the visceral pleura without an area showing a mosaic distribution; (6) a diffuse distribution showed GGO area that homogenously spread and reached the visceral pleura without an area showing a mosaic distribution; (7) consolidation was an area of increased attenuation with obscuration of the underlying vascular markings; (8) centrilobular nodules were ill-defined small nodules that did not extend into the pleural, fissural, or septal surfaces; (9) bronchiectasis showed a bronchus that was locally dilated with the bronchoarterial ratio > 1 (i.e. its internal diameter divided by the diameter of its accompanying artery); (10) a cyst showed the presence of a thin-walled (less than 2-mm thick), well-defined, and circumscribed air-containing lesion with a diameter of 1 cm or more; (11) lymphadenopathy showed a lymph node with a short diameter of more than 1 cm.

### Statistical analysis

Categorical variables are described as counts and percentages. Continuous variables are presented as the median and interquartile range. To detect differences between groups, we used a Fisher’s exact test for categorical variables, and the Mann–Whitney U test for continuous variables. We estimated the overall survival (OS) using a Kaplan-Meier analysis [15]. Differences between survival curves were tested for statistical significance using the two-tailed log-rank test. Univariate and multivariate Cox regression analyses were performed to independently predict OS. To assess the predictive factors for OS, clinical factors such as the HRCT findings were included. BAL findings were excluded from analysis because BAL was not performed in 16 patients (26.2%). A variable was removed from the multivariate model, if the corresponding *P* value was > 0.05 in the univariate model. In the multivariate analysis, a stepwise backward procedure was used to derive the final model of the variables that had a significant independent association with mortality. All statistical analyses were performed using the statistical software R version 2.13.1 (R Foundation for statistical computing, Vienna, Austria). All *P* values are two-tailed, and P values less than 0.05 were considered statistically significant.

## Results

### Patient characteristics

A total of 61 patients were included in our study. Baseline patient characteristics and laboratory data at diagnosis of PjP are shown in Table 1. The median age was 69 years (63–75 years). Thirty-six patients (59.0%) had autoimmune or collagen vascular diseases, and 20 patients (32.8%) had malignancies. A total of 49 patients (80.3%) used corticosteroids and 20 patients (32.8%) used immunosupressants. A total of 45 patients (73.8%) were diagnosed with PjP via bronchoscopy, and a sputum culture was used for the remaining patients. One patient did not receive treatment for PjP because the patient died before receiving a definite PjP diagnosis. A total of 19 patients (31.3%) died at the hospital.

**Table 1:** Patient characteristics

	*N* = 61	
Age, years	69	(63–75)
Gender, female	33	(54.1%)
Ever-smokers	26	(42.6%)
Underlying diseases		
Autoimmune or collagen vascular diseases*	36	(59.0%)
Rheumatoid arthritis	24	(39.3%)
Malignancies**	20	(32.8%)
Haematological malignancies	9	(14.8%)
Solid cancers	11	(18.0%)
Renal diseases	6	(9.8%)
Renal transplantation	1	(1.6%)
Corticosteroid use	49	(80.3%)
Immunosuppressants use	20	(32.8%)
Chemotherapeutic agents use	16	(26.2%)
TMP/SMX prophylaxis	2	(3.3%)
Initial symptoms		
Cough	31	(50.8%)
Dyspnoea	38	(62.3%)
Fever	48	(78.7%)
Initial laboratory findings		
PaO2/FiO2 ratio	213	(139–309)
AaDO2, mmHg	49.9	(27.0–221.1)
Alb, g/dL		
WBC, cell/μL	7300	(5600–10,200)
BUN	21	(16.5–30.5)
LDH, IU/L	366	(300–489)
KL-6, IU/μL	565	(374–1036)
β-D-glucan, pg/mL	135.7	(41.4–372.9)
Diagnostic methods		
BAL	45	(73.8%)
Sputum	16	(26.2%)
BALF cytology positive	17	(27.9%)
Treatments		
TMP/SMX	55	(90.2%)
Atovaquone	1	(1.6%)
Pentamidine	3	(4.9%)
Treatment-related factors		
Adjuvant corticosteroid treatments	53	(86.9%)
Mechanical ventilation	4	(6.6%)
Follow-up duration, days	23	(18–32)
In hospital mortality	19	(31.1%)

Abbreviations Alb, albumin; WBC, white blood cells; BUN, blood urea nitrogen; LDH, lactate dehydrogenase; KL-6, Krebs von den Lungen-6; BALF, bronchoalveolar lavage fluids*Autoimmune or collagen vascular diseases include rheumatoid arthritis.**Malignancies include haematological malignancies and solid cancers

### HRCT findings in patients with non-HIV PjP

**Fig. 1:**
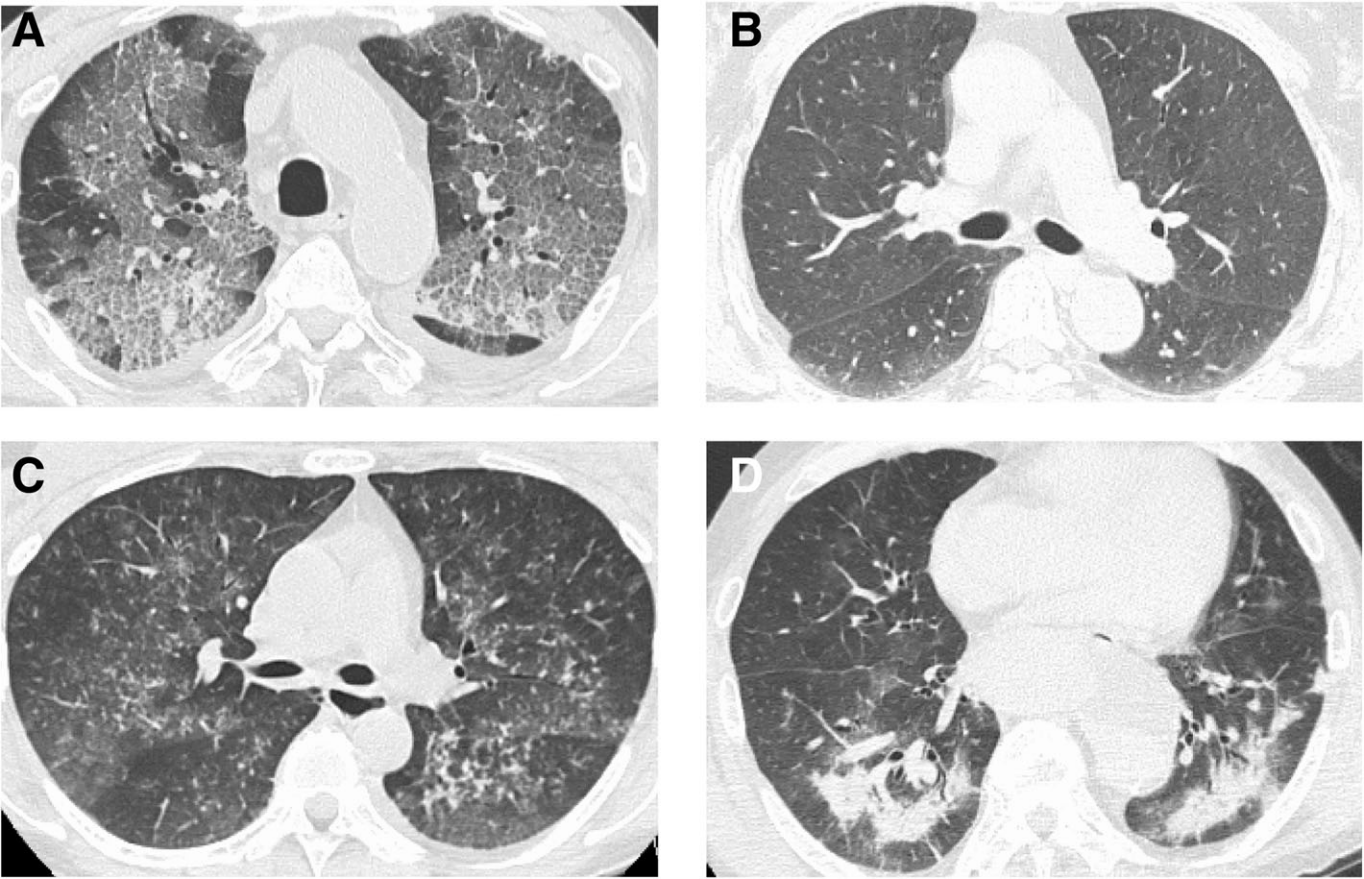
Axial high resolution CT slices of the chest in the lung window of representative four non-HIV cases with proven PjP show crazy paving GGO (**a**), GGO without crazy paving appearance (**b**), centrilobular nodules (**c**), and consolidations (**d**)

The HRCT findings of the patients are shown in Table 2. A total of 29 patients (47.5%) presented with crazy paving GGO; the remaining 32 patients (52.5%) presented without crazy paving GGO. The representative HRCT scans of GGO with and without the crazy paving are shown in Fig. 1a and b, respectively. Centrilobular small nodules were found in 30 patients (49.2%) and consolidations were observed in 23 patients (37.7%). HRCT scans of the representative cases of centrilobular nodules and consolidations are shown in Fig. 1c and d, respectively. Bronchiectasis was observed in 14 patients (23.0%). Cysts or pneumomediastinum were not observed in any patient.

**Table 2:** HRCT findings in patients with non-HIV PjP

HRCT findings	N = 61	
GGO with crazy paving	29	(47.5%)
with sparing periphery	6	(9.8%)
with mosaic distribution	23	(37.7%)
GGO without crazy paving	32	(52.5%)
with sparing periphery	6	(9.8%)
with diffuse distribution	26	(42.6%)
Consolidations	23	(37.7%)
Bronchiectasis	14	(23.0%)
Centrilobular small nodules	30	(49.2%)
Cysts	0	(0.0%)
Intrathoracic lymphadenopathy	3	(4.9%)
Pneumomediastinum	0	(0.0%)

Abbreviations: HRCT, high resolution computed tomography; HIV, human immunodeficiency virus; PjP, *Pneumocystis jirovecii* pneumonia; GGO, ground grass opacity

### Comparison of the characteristics of patients with or without crazy paving GGO

The characteristics of patients with or without crazy paving GGO are shown in Table 3. Patients with crazy paving GGO were more likely to be older than those without crazy paving GGO. The in-hospital mortality of patients with crazy paving GGO was significantly higher than that of patients without crazy paving GGO. The P/F ratios and serum albumin (Alb) levels of patients with crazy paving GGO were significantly lower than in those without. The alveolar-arterial oxygen gradient (AaDO2), LDH, and KL-6 levels in the patients with crazy paving GGO were significantly higher than those without.

**Table 3:** Characteristics of patients with or without crazy paving GGO

	GGO with crazy paving, *N* = 29		GGO without crazy paving, *N* = 32		*P*-value
Age, years	70	(67–76)	67	(51–73)	0.06
Gender, female	18	(62.1%)	15	(46.9%)	0.31
Ever-smokers	10	(34.5%)	16	(50.0%)	0.41
Underlying diseases					
Autoimmune or collagen vascular diseases*	17	(58.6%)	19	(59.4%)	0.99
Rheumatoid arthritis	11	(37.9%)	13	(40.6%)	0.99
Malignancies**	11	(39.7%)	9	(28.1%)	0.59
Haematological malignancies	3	(10.3%)	6	(18.8%)	0.48
Solid cancers	8	(27.6%)	3	(9.4%)	0.10
Renal diseases	1	(3.4%)	5	(15.6%)	0.20
Renal transplantation	0	(0.0%)	1	(3.1%)	0.99
Corticosteroid use	25	(86.2%)	24	(75.0%)	0.34
Immunosuppressants use	8	(27.6%)	12	(37.5%)	0.43
Chemotherapeutic agents use	9	(31.0%)	7	(21.9%)	0.56
TMP/SMX prophylaxis	0	(0.0%)	2	(6.3%)	0.49
Initial symptoms					
Cough	16	(55.1%)	15	(46.9%)	0.31
Dyspnoea	21	(72.4%)	17	(53.1%)	0.19
Fever	24	(82.8%)	24	(75.0%)	0.54
Initial laboratory findings					
PaO2/FiO2 ratio	159	(126–206)	292	(226–357)	< 0.01
AaDO2, mmHg	236	(50–369)	33	(25–56)	< 0.01
Alb, g/dL	2.7	(1.0–4.3)	3.2	(1.6–4.9)	< 0.01
WBC, cell/μL	7200	(5600–10,000)	7950	(6088–10,650)	0.39
BUN	22	(17–35)	21	(16–30)	0.64
LDH, IU/L	426	(368–555)	311	(273–389)	< 0.01
KL-6, IU/μL	831	(522–1117)	439	(326–711)	< 0.01
β-D-glucan, pg/mL	101	(41–373)	147	(55–404)	0.68
Diagnostic methods					
BAL	20	(69.0%)	25	(78.1%)	
BALF cytology positive	11	(37.9%)	6	(18.8%)	
Treatments					
TMP/SMX	27	(93.1%)	28	(87.5%)	0.24
Atovaquone	0	(0.0%)	1	(3.1%)	
Pentamidine	0	(0.0%)	3	(9.4%)	
Treatment-related factors					
Adjuvant corticosteroid treatments	26	(89.7%)	27	(84.4%)	0.71
Mechanical ventilation	3	(10.3%)	1	(3.1%)	0.34
Follow-up duration, days	25	(15–38)	21.5	(19–28)	0.51
In hospital mortality	17	(58.6%)	2	(6.2%)	< 0.01

Abbreviations: Alb, albumin; WBC, white blood cells; BUN, blood urea nitrogen; LDH, lactate dehydrogenase; KL-6, Krebs von den Lungen-6; BALF, bronchoalveolar lavage fluids*Autoimmune or collagen vascular diseases include rheumatoid arthritis.**Malignancies include haematological malignancies and solid cancers

### Overall survivals of the patients with or without crazy paving GGO

**Fig. 2:**
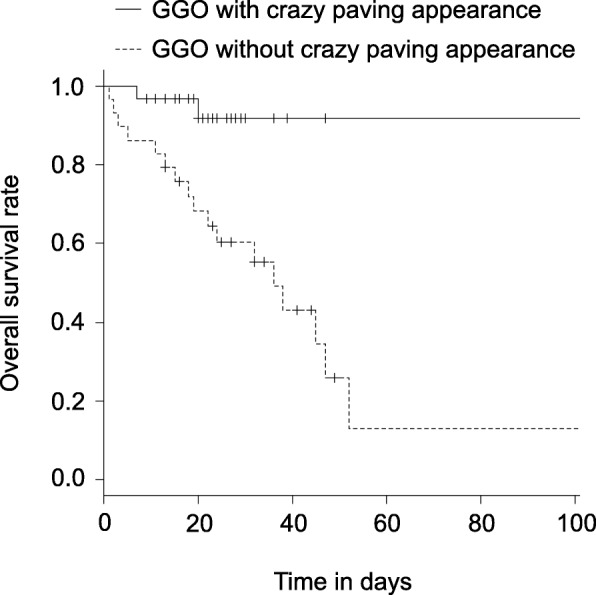
The Kaplan-Meier plots of OS of the groups with or without crazy paving GGO are shown

We compared the OS between the patients with and without crazy paving GGO. The patients with crazy paving GGO experienced a significantly worse OS than the patients without (*P* < 0.01) (Fig. 2). The 30-day OS and 90-day OS were 60.3 and 12.9%, respectively, for the patients with crazy paving GGO, and 91.8 and 91.8%, respectively, for the patients without crazy paving GGO.

### Prognostic impact of clinical factors on mortality

A univariate Cox regression analyses identified prognostic factors, including the P/F ratios (hazard ratio [HR], 0.99; 95% confidence interval [95% CI], 0.99–0.99; *P* < 0.01), serum Alb levels (HR, 0.23; 95% CI, 0.11–0.50; *P* < 0.01), crazy paving GGO (HR, 9.17; 95% CI, 1.82–46.1; *P* < 0.01), consolidations (HR, 11.2; 95% CI, 2.62–48.1; *P* < 0.01), and bronchiectasis (HR, 10.0; 95% CI, 2.40–42.0; *P* < 0.01) (Additional file 1: Table S1). A multivariate Cox regression analysis showed that serum Alb levels (HR, 0.20; 95% CI, 0.07–0.58; *P* < 0.01) and crazy paving GGO (HR, 10.8; 95% CI, 1.40–83.0; *P* = 0.02) were significant independent prognostic factors (Table 4).

**Table 4:** Multivariate analysis for mortality

Variables	HR	95% CI	*P*-value
Alb	0.20	0.07–0.58	< 0.01
GGO with crazy paving	10.8	1.40–83.0	0.02

Abbreviations: Alb, albumin; GGO, ground grass opacity; HR, hazard ratio; 95% CI, 95% confidence interval; NA, not applicable

## Discussion

Our study describes the relevance of HRCT findings in 61 non-HIV PjP patients from a multicentre cohort, one of the largest cohorts ever reported. All patients presented with GGO, and 29 patients (47.5%) presented with crazy paving GGO. We show that compared to patients without, patients with crazy paving GGO have significantly lower P/F ratios and serum Alb levels, and significantly higher levels of laboratory markers reflecting lung injury, such as serum KL-6 levels and serum LDH levels. We also show the independent unfavourable prognostic impact of crazy paving GGO and low serum Alb levels. Our study is the first to show the prognostic impact of HRCT findings during the diagnosis of non-HIV PjP.

Extensive GGO are the main findings in patients with non-HIV PjP [7, 10]. Several patterns of GGO distribution have been described, such as a central distribution with relative peripheral sparing, a mosaic pattern, and a diffuse and nearly homogeneous distribution. Notably, some patients with non-HIV PjP present septal lines with or without intralobular lines superposed on GGO (i.e. crazy paving GGO) [7, 10–12]. The GGO distributions we observed included a peripheral sparing distribution (12 patients, 19.6%), a mosaic distribution (23 patients, 37.7%), and a diffuse distribution (26 patients, 42.6%). In addition, 29 patients (47.5%) had crazy paving GGO. Moreover, all patients with GGO with a mosaic distribution also had crazy paving GGO. Consolidations and bronchiectasis are reported to appear in only advanced disease stages [12]. In our study, 23 patients (37.7%) exhibited consolidations and 14 patients (23.0%) exhibited bronchiectasis. Centrilobular small nodules are often observed in non-HIV PjP patients [7, 10] and were found in 30 of our patients (49.2%). Cysts and intrathoracic lymphadenopathy are uncommon findings in non-HIV PjP [7, 10] and were seldom observed our investigation.

Studies on an association between chest HRCT findings and clinical features are limited in patients with PjP that are either HIV-positive or -negative. Tokuda et al. described three radiographic patterns on HRCT images in patients with PjP, rheumatoid arthritis and HIV infection [16]. No significant correlation was observed between radiographic patterns and clinical features, including clinical signs, laboratory markers, and death. Tasaka et al. assessed HRCT findings in patients with PjP, malignancy, and HIV infection [4]. In addition, they scored the extent of GGO and consolidation in relatively small regions in the lung fields, but did not identify any significant correlation between HRCT findings and laboratory markers, such as the oxygenation index and serum levels of KL-6, LDH, and CRP. Chou et al. measured mean lung attenuation (MLA) and the extent of increased attenuation (EIA) of PjP lesions on chest HRCT images from 40 patients with PjP that were HIV-negative [17]. MLA and EIA of PjP lesions were significantly associated with the P/F ratio, acute physiology, and chronic health evaluation (APACHE) II score in intensive care unit, and assisted mechanical ventilation; however, MLA and EIA of PjP lesions had limited value in identifying survivors and non-survivors. Our study focused on the relationship between clinical outcomes and the characteristics of GGO with or without crazy paving. The crazy paving appearance on chest HRCT imaging shows scattered GGO with superimposed interlobular septal thickening and intralobular lines [18]. Initially described in cases of alveolar proteinosis [19, 20], this pattern has subsequently been reported in a variety of infectious, neoplastic, idiopathic, inhalational, and sanguineous disorders of the lung [18]. PjP is representative of an infectious disorder that is associated with crazy paving GGO [21, 22], and the importance crazy paving GGO has not been investigated. We show that the background patient characteristics were not significantly different between patients with and without crazy paving GGO. In addition, compared to those without, patients with crazy paving GGO, serum KL-6 and LDH levels were significantly higher and the P/F ratio and serum Alb levels were significantly lower. High serum KL-6 and LDH levels reflect lung injury [23–26]; therefore, our results suggested that crazy paving GGO on HRCT findings may reflect acute lung injury, and that patients with crazy paving GGO are more likely to experience severe respiratory failure and death compared to those without crazy paving GGO.

Several prognostic factors of non-HIV PjP have been reported [27, 28]. Kim et al. showed that AaDO2, combined bacteraemia, (blood urea nitrogen) BUN, and chronic lung diseases are independent prognostic factors [27]. In our study, we excluded patients with other concurrent infections, or pulmonary comorbidities such as interstitial pneumonia or lung cancer, and AaDO2 and BUN were not significant prognostic factors. Weng et al. demonstrated that age, white blood cell counts, and pneumomediastinum were independently associated with hospital mortality in patients with non-HIV severe PjP in an intensive care unit. We did not detect pneumomediastinum in any of our patients. Moreover, age and white blood cell counts were not associated with mortality. Differences in study population of our study from that of previous studies could account for differences in the association of mortality and prognostic factors such as AaDO2, BUN, age, and white blood cell counts. We show that the serum albumin level is a significant independent poor prognosis factor. This result is consistent with the results of previous studies of pneumonia that demonstrate that hypoalbuminaemia is a prognostic marker in PjP patients with autoimmune diseases or collagen vascular diseases [29, 30]. Overall, these results suggest that treatment strategies for HIV-negative patients with PjP should consider the serum Alb level and crazy paving GGO appearance on HRCT findings at diagnosis because these observations indicate a poor prognosis.

The present study has some limitations. First, our study was retrospective; thus, our results should be validated in a prospective multicentre investigation. Second, we did not evaluate the impact of BAL findings on survival because BAL procedures were not performed in approximately 30% of patients mainly due to respiratory failure. However, previous studies show that neutrophilia in BALF is an independent prognostic marker [31, 32]. Third, some patients were diagnosed with PjP based on the results of PCR. PCR based detection of *P. jirovecii* could possibly be a false positive result. However, our diagnosis criteria for PjP included clinical symptoms that are suggestive of a lower respiratory tract infection, new pulmonary infiltrates on HRCT images, and significantly elevated plasma β-D-glucan to exclude false positive results.

## Conclusions

In our multicentre investigation of the HRCT findings in 61 HIV-negative patients with PjP, we show that crazy paving GGO and a low serum Alb level are independent risk factors for mortality. Further prospective studies should be performed to confirm our findings.

## Additional file

Additional file 1: **Table S1.** Univariate analysis for mortality. A univariate Cox regression analyses were employed to identify significant prognostic factors. (DOCX 15 kb)

## Abbreviations

AaDO2: alveolar-arterial oxygen gradient
APACHE: acute physiology, and chronic health evaluation
BAL: bronchoalveolar lavage
BALF: bronchoalveolar lavage fluid
BUN: blood urea nitrogen
CMV: cytomegalovirus
CT: computed tomography
EIA: extent of increased attenuation
GGO: ground grass opacities
HIV: human immunodeficiency virus
HRCT: high-resolution computed tomography
KL-6: Krebs von den Lungen-6
LDH: lactate dehydrogenase
OS: overall survival
P/F: PaO_2_/FiO_2_
PjP: *Pneumocystis jirovecii* pneumonia
TMP/SMX: trimethoprim/sulfamethoxazole; β-D-glucan, (1–3)-β-D-glucan
