# Supplementary material for: Prognostic significance of crazy paving ground grass opacities in non-HIV Pneumocystis jirovecii pneumonia: an observational cohort study
Source: BMC Pulm Med. 2019 Feb 21;19:47. doi: 10.1186/s12890-019-0813-y (PMC6385404; doi:10.1186/s12890-019-0813-y)
Supplement: Supplementary file 1 — Table S1. Univariate analysis for mortality. A univariate Cox regression analyses were employed to identify significant prognostic factors. (DOCX 15 kb) [file 12890_2019_813_MOESM1_ESM.docx]

| Supplementary Table. Univariate analysis for mortality | | | |
| --- | --- | --- | --- |
| Variables | HR | 95% CI | P-value |
| Age | 1.00 | 0.97-1.04 | 0.91 |
| Female | 0.51 | 0.18-1.45 | 0.20 |
| Ever-smokers | 1.37 | 0.50-3.74 | 0.54 |
| Underlying diseases |  |  |  |
| Autoimmune or collagen vascular diseases | 0.84 | 0.33-2.13 | 0.72 |
| Rheumatoid arthritis | 0.42 | 0.15-1.19 | 0.10 |
| Malignancies | 1.60 | 0.63-4.10 | 0.32 |
| Haematological malignancies | 0.82 | 0.19-3.59 | 0.79 |
| Solid cancers | 2.07 | 0.75-5.73 | 0.16 |
| Renal diseases | 2.13 | 0.77-5.90 | 0.15 |
| Renal transplantation | NE |  |  |
| Corticosteroid use | 3.52 | 0.47-26.6 | 0.22 |
| Immunosuppressants use | 0.40 | 0.13-1.22 | 0.11 |
| Chemotherapeutic agents use | 1.92 | 0.73-5.05 | 0.18 |
| TMP/SMX prophylaxis | 2.94 | 0.38-23.0 | 0.30 |
| Initial symptoms |  |  |  |
| Cough | 1.54 | 0.62-3.85 | 0.36 |
| Dyspnoea | 2.57 | 0.84-7.86 | 0.10 |
| Fever | 1.08 | 0.30-3.80 | 0.91 |
| Initial laboratory findings |  |  |  |
| PaO2/FiO2 ratio | 0.99 | 0.99-0.99 | <0.01 |
| AaDO2 | 1.00 | 1.00-1.00 | 0.20 |
| Alb | 0.23 | 0.11-0.50 | <0.01 |
| WBC, cell/µL | 1.00 | 1.00-1.00 | 0.16 |
| BUN | 1.04 | 0.99-1.08 | 0.06 |
| LDH, IU/L | 1.00 | 1.00-1.00 | 0.14 |
| KL-6, IU/µL | 1.00 | 1.00-1.00 | 0.20 |
| β-D-glucan, pg/mL | 1.00 | 1.00-1.00 | 0.86 |
| Diagnostic methods |  |  |  |
| BALF | 1.70 | 0.67-4.35 | 0.27 |
| BALF cytology positive | 1.49 | 0.50-4.45 | 0.48 |
| Treatments |  |  |  |
| TMP/SMX | NE |  |  |
| Atovaquone | NE |  |  |
| Pentamidine | NE |  |  |
| Treatment-related factors |  |  |  |
| Adjuvant corticosteroid treatments | 0.60 | 0.13-2.75 | 0.51 |
| Mechanical ventilation | 1.38 | 0.31-6.11 | 0.67 |
| HRCT findings |  |  |  |
| GGO with crazy paving | 9.17 | 1.82-46.1 | <0.01 |
| Consolidations | 11.2 | 2.62-48.1 | <0.01 |
| Bronchiectasis | 10.0 | 2.40-42.0 | <0.01 |
| Centrilobular small nodules | 0.86 | 0.25-2.90 | 0.81 |
| Abbreviations: Alb, albumin; WBC, white blood cells; BUN, blood urea nitrogen; LDH, lactate dehydrogenase; KL-6, Krebs von den Lungen-6; BALF, bronchoalveolar lavage fluids, HRCT, high resolution computed tomography; GGO, ground grass opacity; HR, hazard ratio; 95% CI, 95% confidence interval; NE, not evaluable *Autoimmune or collagen vascular diseases include rheumatoid arthritis. **Malignancies include haematological malignancies and solid cancers. | | | |
